# Controlling Oral Polymicrobial Biofilm Using Usnic Acid on the Surface of Titanium in the Artificial Saliva Media

**DOI:** 10.3390/antibiotics14020115

**Published:** 2025-01-22

**Authors:** Nazia Tabassum, Fazlurrahman Khan, Geum-Jae Jeong, Do Kyung Oh, Young-Mog Kim

**Affiliations:** 1 Marine Integrated Biomedical Technology Center, The National Key Research Institutes in Universities, Pukyong National University, Busan 48513, Republic of Korea; nazia99@pukyong.ac.kr (N.T.); dkoh@pknu.ac.kr (D.K.O.); 2 Research Center for Marine Integrated Bionics Technology, Pukyong National University, Busan 48513, Republic of Korea; 3 Ocean and Fisheries Development International Cooperation Institute, Pukyong National University, Busan 48513, Republic of Korea; 4 International Graduate Program of Fisheries Science, Pukyong National University, Busan 48513, Republic of Korea; 5 Department of Food Science and Technology, Pukyong National University, Busan 48513, Republic of Korea

**Keywords:** titanium dental implants, polymicrobial diseases, usnic acid, antibiofilm, human artificial saliva, treatment strategy

## Abstract

**Background/Objectives:** Titanium dental implants, while highly successful, face challenges due to polymicrobial infections leading to peri-implantitis and implant failure. Biofilm formation on implant surfaces is the primary cause of these infections, with factors such as matrix production and cross-kingdom interactions contributing to the microbial accumulation of bacterial and fungal pathogens species. To combat this issue, naturally derived molecules have been reported to overcome the hurdle of antimicrobial resistance against the application of conventional antibiotics and antifungals. **Methods:** The present study aimed to employ the lichen-derived molecules, usnic acid (UA), to retard the development of biofilms of bacterial and fungal pathogens on the surface of titanium kept in the human artificial saliva (HAS) working as a growth-supporting, host-mimicking media. **Results:** The minimum inhibitory concentration of UA in HAS towards *Candida albicans* was >512 µg/mL, whereas against *Staphylococcus aureus* and *Streptococcus mutans*, it was determined to be 512 µg/mL. Whereas, in the standard growth media, the MIC value of UA towards *S. mutans* and *S. aureus* were 8 and 16 µg/mL; however, against *C. albicans*, it was 512 µg/mL. UA synergistically enhanced the efficacy of the antibiotics toward bacterial pathogens and the efficacy of antifungals against *C. albicans*. The antibiofilm results depict the fact that in the HAS, UA significantly reduced both mono-species of *S. mutans*, *S. aureus*, and *C. albicans* and mixed-species biofilm of *C. albicans* with *S. mutans* and *S. aureus* on the surface of the titanium. **Conclusions:** The present study showed that UA is a promising natural drug that can control oral polymicrobial disease as a result of the application of dental implants.

## 1. Introduction

Oral polymicrobial biofilms play a crucial role in diseases related to dental caries and periodontics, the two most prevalent microbial-induced disorders globally [1,2]. These complex communities of bacterial and fungal species form highly organized structures on tooth surfaces embedded within an extracellular matrix [3]. The assembly and function of oral microbial communities are regulated by sophisticated signaling systems and influenced by host and environmental factors [4]. Dental implants, while generally successful, can be susceptible to polymicrobial infections leading to peri-implantitis and potential implant failure [5]. The microbiota associated with infected implants resembles that of chronic periodontitis, primarily consisting of anaerobic Gram-negative bacteria such as *Porphyromonas gingivalis* and *Prevotella intermedia* [6,7]. Recent evidence suggests that *Candida albicans*, an opportunistic fungal pathogen, may significantly enhance implant infectious growth by establishing cross-kingdom interactions with other oral bacteria such as *Streptococcus mutans* and *Staphylococcus aureus* [8,9,10]. Polysaccharides derived by *C. albicans* augment *S. mutans* cells to adhere both in vitro and in vivo, enhancing mixed biofilm architecture [11]. Similarly, *C. albicans* facilitates *S. aureus* biofilm formation in serum, providing scaffolding and matrix coating, which increases *S. aureus* resistance to vancomycin [12].

These polymicrobial interactions contribute to oral diseases, pose challenges for conventional treatments, and serve as reservoirs for antibiotic resistance [13,14]. These biofilms exhibit increased resistance to antimicrobials, with bacteria in biofilms being up to 10,000 times less sensitive to antibiotics compared to planktonic bacteria [15]. The formation of oral biofilms involves multiple stages and adapts to environmental changes, making their clinical management challenging [16]. Conventional antibiotics and antifungal drugs often fail to penetrate biofilms effectively, leading to treatment failures [16]. Furthermore, the application of antimicrobials for infection treatment can also cause significant side effects, particularly in vulnerable populations such as the elderly and critically ill patients [17,18]. These adverse effects include neurotoxicity, nephrotoxicity, and disruption of the microbiome [19]. Careful monitoring and dose adjustments are essential to minimize risks [20]. Furthermore, most of the antibiofilm drugs that have been evaluated for their efficacy in the standard growth media used in laboratory settings often fail to accurately represent in vivo conditions, potentially leading to ineffective treatments when applied in host systems [21]. Hence, it has been reported that the complex nature of these polymicrobial biofilms, combined with the irregular topography of implant surfaces, makes treatment challenging [5].

Currently, there is no consensus on the best clinical protocol for controlling microbial accumulation on dental implants or treating peri-implant diseases [5,7]. However, new approaches, including natural and synthesized drugs, show promise in managing these complex biofilms [22]. Marine organisms are a rich source of bioactive compounds ranging from simple peptides to complex alkaloids and terpenes with antimicrobial properties, offering potential solutions to combat drug-resistant pathogens and biofilm-associated infections [23,24]. Usnic acid, a lichen-derived secondary metabolite, has gained attention as a promising antimicrobial agent with a broad spectrum of activity [25,26,27]. Its mechanism of action involves inhibiting RNA synthesis, disrupting DNA replication, and destroying microbial membranes [28,29]. Studies have shown that usnic acid effectively reduces biofilm formation and virulence factors in *C. albicans*, including hyphal growth and adhesion [30,31]. It also inhibits biofilm formation by *S. aureus* and alters *Pseudomonas aeruginosa* biofilm morphology when incorporated into polymer surfaces [32]. In addition to its antimicrobial properties, usinic acid exhibits favorable pharmacokinetic and safety profiles, further supporting its potential as a therapeutic agent [26]. Studies have indicated minimal cytotoxicity towards mammalian cells at therapeutic concentrations, underscoring its potential for clinical application [33].

To effectively treat biofilm infections, a multidisciplinary approach involving biofilm-active antimicrobials, combination therapies, and strategies to enhance drug penetration is crucial [34,35]. The first objective of the present study is to determine the minimum inhibitory concentration of usnic acid against *C. albicans*, *S. aureus*, and *S. mutans*, followed by examining the synergy effect of usnic acid in combination with the conventional antibiotics and antifungal against these pathogens. The second objective of this study is to examine the antibiofilm efficacy of the usnic acid towards the single- and polymicrobial species biofilm of *C. albicans*, *S. aureus*, and *S. mutans*. The biofilm inhibition properties of usnic acid were evaluated using a titanium surface, since titanium implants have been best used in dental and joint implants for their biocompatibility and favorable physio-chemical properties [36,37,38]. In this experiment, human artificial saliva was selected as a close replica of the host oral environment to study growth and controlled testing of the anti-biofilm effect of usnic acid on *C. albicans*, *S. aureus*, and *S. mutans* biofilms on the surface of the titanium surface. It has been reported that the formulations of artificial saliva typically contain electrolytes, enzymes, proteins, and antimicrobial agents, replicating key components of natural saliva [39,40]. Artificial saliva provides a standardized environment for studying oral microbial growth, biofilm formation, and interactions between different species [41,42]. It can be used to investigate the effects of various factors on oral health, including pH, antimicrobial properties, and lubrication [43,44].

## 2. Results and Discussion

### 2.1. Minimum Inhibitory Concentration Toward Oral Pathogens

Usnic acid, a lichen secondary metabolite, demonstrated potent antifungal activity against various *Candida* species, including *C. albicans*. Studies have shown usnic acid exhibit minimum inhibitory concentration (MIC) values ranging from 0.0008 to 0.5 mg/mL against different microorganisms [45]. The MIC values of usnic acid towards bacterial pathogens (e.g., *S. aureus* and *S. mutans*) and fungal pathogens (e.g., *C. albicans*) were examined in the human artificial saliva. The MIC value of usnic acid towards *C. albicans* was >512 µg/mL, whereas the MIC value against *S. aureus* and *S. mutans* was determined to be 512 µg/mL. The MIC values of usnic acid examined in the standard growth media, such as TSB (for bacteria) and PDB (for *C. albicans*), were different as observed in artificial saliva. The MIC values of usnic acid against *S. mutans*, *S. aureus*, and *C. albicans* were 8 µg/mL, 16 µg/mL, and 512 µg/mL, respectively (Table 1). Several reports previously reported different MIC values of usnic acid towards different species of bacterial pathogens. Priya et al. [46] reported the MIC value of usnic acid against *S. mutans*, with the value of 5 μg/mL, which is almost similar to the present study. The MIC of UA-loaded liposomes against *S. aureus* ranged from 8 to 16 μg/mL, while free UA was 8 μg/mL [47], which is two-fold lower than observed in the present study, using only free usnic acid against *S. aureus*. This indicates that the application of usnic acid on liposomal encapsulation and polymer complexation results in enhanced antimicrobial efficacy [47,48]. When complexed with polyacrylamides, usnic acid showed enhanced antimicrobial activity against *Staphylococcus epidermidis*, with lower MICs compared to the free drug [48]. However, the MIC value of free usnic acid against *S. aureus* and *C. albicans*, as reported earlier, was found to be 0.125 mg/mL (125 µg/mL) [45], which is four-fold lower, as determined in the present study against *C. albicans*.

### 2.2. Synergistic Effects of Usnic Acid with Antibiotics and Antifungal

Polymicrobial biofilms pose significant challenges in treating infections due to their complex interactions and enhanced resistance to antimicrobials [49,50]. Conventional antibiotics often proved insufficient in eradicating biofilm infections, necessitating alternative strategies [34,51]. The combination therapies using antibiotics and antifungals with natural/synthetic molecules or nanomaterials have shown promise in combating mixed bacterial-fungal biofilms [50,52]. Synergistic effects have been observed when combining gentamicin with benzylpenicillin or rifampicin against streptococcal biofilms [53]. Antimicrobial peptides have emerged as potential broad-spectrum agents against biofilms, demonstrating synergy with antibiotics and targeting stress responses in bacteria [54,55]. Phage therapy combined with antibiotics has also shown enhanced efficacy against mono and dual-species biofilms [56]. Furthermore, the antibiofilm peptide 1018 has demonstrated synergistic interactions with various antibiotics against multidrug-resistant pathogens [57]. Combinations of fosfomycin, ciprofloxacin, and gentamicin have exhibited synergistic activity against *E. coli* and *P. aeruginosa* biofilms [58]. In the present study, we selected these antibiotics (e.g., streptomycin, tetracycline, rifampicin, gentamicin, and ciprofloxacin) and antifungal (fluconazole, and amphotericin B) to perform the synergy with usnic acid against the *S. mutans*, *S. aureus*, and *C. albicans* which are well reported to form a polymicrobial biofilm [52].

Table 1 shows the combined action of usnic acid with antibiotics and antifungals on *S. mutans*, *S. aureus*, and *C. albicans*. The combined minimum inhibitory concentration (MIC) values were significantly reduced across all tested microorganisms. For *S. mutans* the combined MIC was reported to be between 1 µg/mL and 0.008 µg/mL, for *S. aureus* between 1 µg/mL and 0.125 µg/mL, and for *C. albicans* between 4 µg/mL and 2 µg/mL. The reduction suggests that the combination therapy enhanced the antimicrobial efficacy. Furthermore, the fractional inhibitory concentration (FIC) indices of the combined treatment of usnic acid and standard antimicrobial agents were consistently <0.5. Similarly, the summation of FIC (ΣFIC) for all the tested microorganisms remained to be <0.5, which means the synergistic effect of combination therapy. This synergy suggests that the combination therapy of usnic acid with antibiotics/or antifungals enhances the effectiveness of the treatment, potentially lowering the dosage requirement and thus reducing the risk of resistance. Such synergy effects between natural compounds like usnic acid and conventional antibiotics have previously been reported in gram-negative bacteria [59]. These results align with the concept that combining natural products with conventional antibiotics/antifungals can enhance the overall efficacy of treatments against resistant bacteria like *S. aureus* and fungal pathogens like *C. albicans* [60]. The combination therapy synergistic result of antibiotics with usnic acid is reported [61]. Previous studies showed that usnic acid has the least resistance towards *S. aureus* but the most resistance towards gram-negative bacteria like *P. aeruginosa* and *E. coli* [62].

### 2.3. Inhibition of Biofilm on the Surface of Titanium in Saliva Media

**Figure 1:**
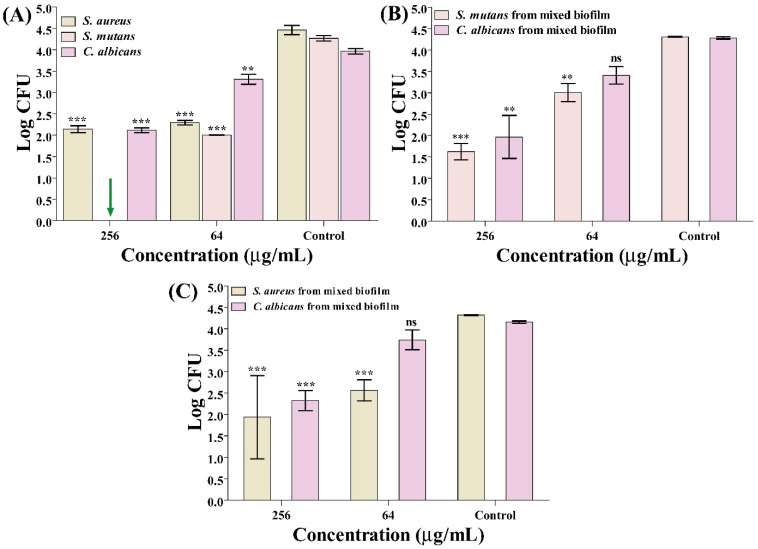
Inhibition of the growth of *C. albicans*, *S. aureus*, and *S. mutans* mono- and mixed-species biofilms on a titanium surface in saliva. (**A**) CFU of cells of *S. aureus*, *S. mutans*, and *C. albicans*; (**B**) CFU of mixed-species biofilms including *S. mutans* and *C. albicans*; and (**C**) CFU of mixed-species biofilms containing *S. aureus* and *C. albicans*. Green arrow is complete inhibition, and ns means no significance. *** *p* < 0.0001 and ** *p* < 0.01 were considered significant, and the bar graph was plotted as mean log values of CFU with an error bar showing the standard deviation of CFUs.

In the monoculture, *S. aureus* showed nearly equal log reductions of (2.32 ± 0.17) and (2.17 ± 0.14) with inhibition percentages of 52% and 48.5% in both 256 µg/mL concentrations and 64 µg/mL to that of the control group. On the other hand, *S. mutans* were completely 100% inhibited at high concentrations, whereas there was 53% inhibition with a log reduction of (2.26 ± 0.11) at low concentrations (Figure 1A). In comparison, *C. albicans* biofilm showed a log reduction of (1.85 ± 0.12) and inhibition of 46.6% at 256 µg/mL and a log reduction of (0.65 ± 0.23), which was 16.5% at 64 µg/mL concentration. For the co-culture of *S. mutans* and *C. albicans*, a log reduction value of (2.69 ± 0.35) was obtained in *S. mutans* biofilm, which equaled an inhibition of 62.3%, whereas a log reduction of (1.30 ± 0.36) and inhibition of 30.2% was present at both concentrations (Figure 1B). In comparison, *C. albicans* biofilm had a respective log reduction of (2.32 ± 0.88) and (0.8 ± 0.35) at both concentrations and inhibition percentages of 54% and 20.44% (Figure 1B). On the other hand, in co-culture of *S. aureus* and *C. albicans*, inhibition for *S. aureus* biofilms was recorded at a log reduction of (2.38 ± 1.66) and an inhibition percentage of 55.1% in 256 µg/mL and a log reduction of (1.76 ± 0.41) with a 40.68% inhibition in 64 µg/mL concentrations (Figure 1C). In contrast, for *C. albicans*, a log reduction of (1.83 ± 0.36) was measured, which was 44% at 256 µg/mL and only a small log reduction of (0.41 ± 0.45) or 9.9% inhibition at 64 µg/mL (Figure 1C).

Previous reports showed that it exhibits potent activity against *S. mutans*, with a MIC of 5 µg/mL, inhibiting biofilm formation and downregulating virulence-associated genes [46]. As previously reported, usnic acid can effectively inhibit biofilm formation and virulent morphological traits in *C. albicans*, reducing biofilm thickness and preventing yeast-to-hyphal transition [30]. Usnic acid also shows strong activity against *C. orthopsilosis* and C. *parapsilosis*, both in planktonic and biofilm conditions, reducing the metabolic activity of sessile cells by 80% [63]. However, the above studies conducted antibiofilm assays of usnic acid against these microbial pathogens in the standard growth media. The significant biofilm inhibition of the usnic acid towards mono- and mixed-species of bacterial and fungal pathogens in the saliva media shows a novel study. When usnic acid is employed in the in vivo system, its antibiofilm effects may replicate the host environment as determined in the artificial saliva [64]. The present findings collectively suggest that usnic acid has the potential as a therapeutic agent against oral polymicrobial infections, particularly those involving *Candida*, *Staphylococcus*, and *Streptococcus* species, by targeting biofilm formation.

### 2.4. Inhibition of Biofilm on the Surface of Titanium in Standard Growth Media

**Figure 2:**
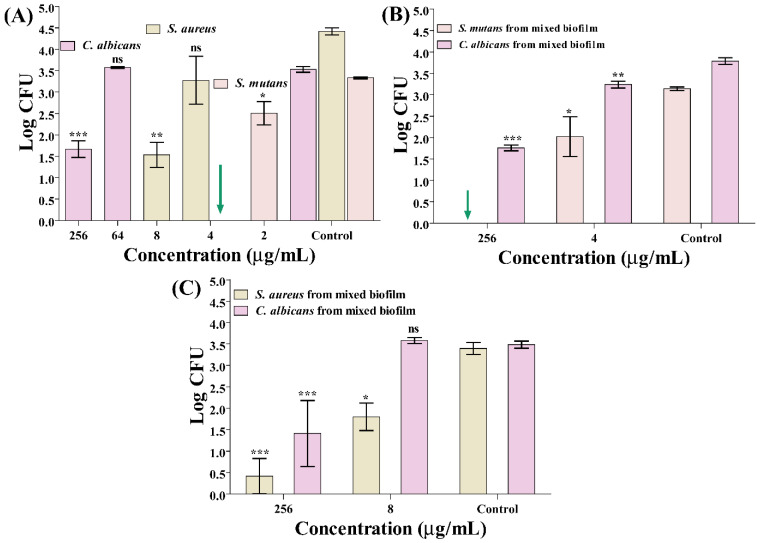
Inhibition of the growth of *C. albicans*, *S. aureus*, and *S. mutans* mono- and mixed-species biofilms on a titanium surface in the standard growth media. (**A**) CFU of cells of *S. aureus*, *S. mutans*, and *C. albicans*; (**B**) CFU of mixed-species biofilms including *S. mutans* and *C. albicans*; and (**C**) CFU of mixed-species biofilms containing *S. aureus* and *C. albicans*. Green arrow is complete inhibition, and ns means no significance. *** *p* < 0.0001, ** *p* < 0.01, and * *p* < 0.05 were considered significant, and the bar graph was plotted as mean log values of CFU with an error bar showing the standard deviation of CFUs.

The antibiofilm activity of usnic acid against mono- and mixed-species of *S. mutans*, *S. aureus*, and *C. albicans* was evaluated at high and low concentrations between 256 µg/mL and 2 µg/mL in the standard growth media. In the mono-species culture of *C. albicans*, the inhibition percentage observed at 256 µg/mL was 52.82 % (log reduction of 1.87 ± 0.38), while nearly zero inhibition at 64 µg/mL with respect to the control group (Figure 2A). For *S. aureus*, biofilm inhibition showed a log reduction of (2.88 ± 0.43), and an inhibition percentage of 65.2% at 8 µg/mL, while significantly low to just 25.8% (log reduction of 1.14 ± 1.04) at 4 µg/mL concentrations (Figure 2A). In *S. mutans*, a drastic inhibition of ~100% of cells (log reduction of 3.33 ± 0.04) was found at 4 µg/mL, whereas it was just 24.8% ((log reduction of 0.83 ± 0.44) at 2 µg/mL (Figure 2A).

In the combined setting of cultures of *S. mutans* and *C. albicans*, *S. mutans* showed complete inhibition ~100% ((log reduction of 3.14 ± 0.07) at 256 µg/mL and 35.5% (log reduction of 1.12 ± 0.79) at 4 µg/mL (Figure 2B). At the same time, *C. albicans* showed a log reduction of (2.03 ± 0.02) and an inhibition percentage of 53.5% at 256 µg/mL, which decreased to (0.55 ± 0.26) reduction, i.e.; 14.5% at 4 µg/mL (Figure 2B). Similarly, in the combined culture *S. aureus* and *C. albicans*, inhibition of *S. aureus* was 87.7% (log reduction of 2.98 ± 0.58) at 256 µg/mL and 47.7% (log reduction of 1.6 ± 0.8) at 8 µg/mL, while *C. albicans* exhibited log reduction of (2.07 ± 1.19) and 59.5% inhibition at 256 µg/mL and no inhibition at 8 µg/mL (Figure 2C). These results suggest differential antibiofilm effects depending on the species and concentration, with significant variations observed in combined cultures. It has been found that biofilms composed of polymicrobial species are more resilient than biofilms composed of a single species, which leads to increased resistance to antimicrobial agents [10]. A significant dose of drugs is required to suppress the biofilm composed of multiple species of microorganisms [65].

### 2.5. Microscopic Visualization of the Biofilm on the Surface of Titanium Treated with Usnic Acid

**Figure 3:**
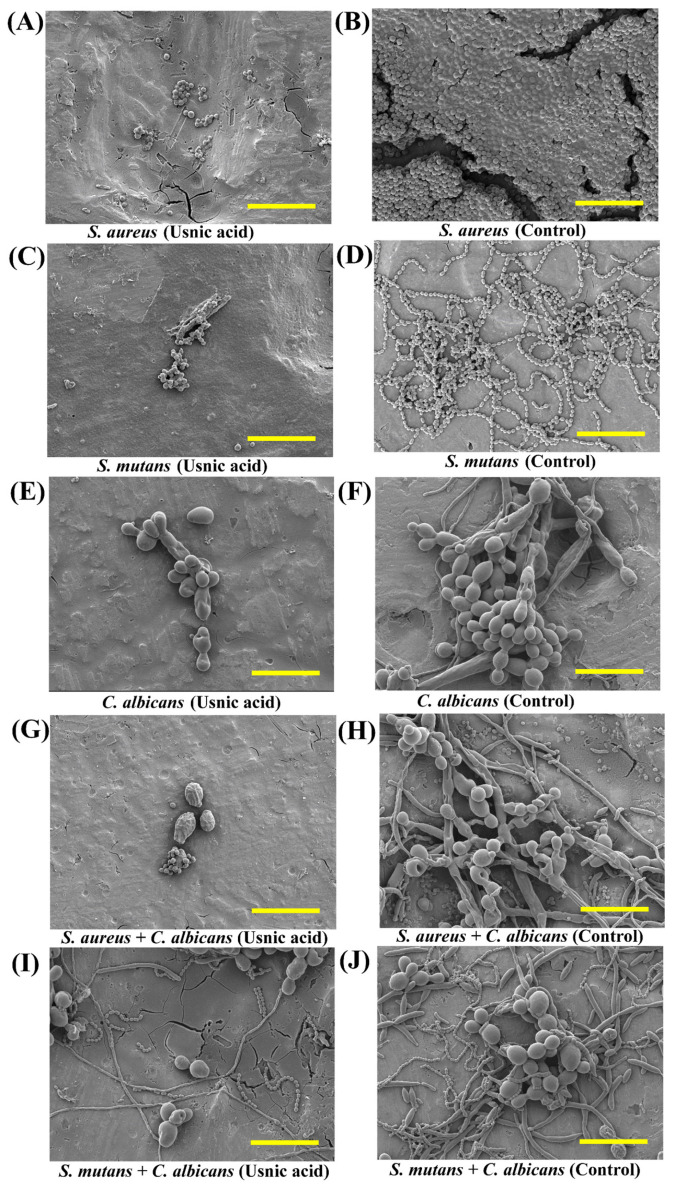
Biofilm architecture of *S. aureus*, *S. mutans*, and *C. albicans* in mono- and mixed-species forms on titanium surface treated with usnic acid. (**A**) Biofilms of *S. aureus* treated with UA; (**B**) Biofilms of *S. aureus* control; (**C**) Biofilms of *S. mutans* treated with UA; (**D**) Biofilm of *S. mutans* control; (**E**) Biofilms of *C. albicans* treated with UA; (**F**) Biofilms of *C. albicans* control; (**G**) Biofilms of *S. aureus* + *C. albicans* treated with UA; (**H**) Biofilms of *S. aureus* and *C. albicans* control; (**I**) Biofilms of *S. mutans* + *C. albicans* treated with UA; and (**J**) Biofilms of *S. mutans* + *C. albicans* control. The yellow scale bars represent 10 µm.

The examination of the biofilm inhibitory effects of usnic acid towards the bacterial and fungal pathogens in the form of mono- and polymicrobial species in the artificial saliva was evaluated by scanning electron microscopy (Figure 3). The attachment of mono-species *S. aureus* and *S. mutans* were found to be significantly inhibited on the surface of the titanium coupons when exposed with sub-MIC of usnic acid (Figure 3A,C). However, the control cells, which are nontreated with usnic, were found to be freely attached in a dense form on a titanium surface (Figure 3B,D). A similar result has also been observed in the case of *C. albicans*, where treated cells are less attached to the titanium surface (Figure 3E). However, the control cells of *C. albicans* form a dense biofilm on the surface, and the cells become filamentous (Figure 3F). Previous studies have also shown that usnic acid inhibits yeast-to-hyphal morphogenesis in *C. albicans* while also lowering biofilm thickness [30]. Inhibiting hyphal growth in *C. albicans* is one of the ways to reduce its virulence and pathogenic qualities [66].

The sub-MIC also greatly affected the biofilm inhibitory effects of usnic acid towards polymicrobial-species cells. The mixed-species biofilm of *S. aureus* and *C. albicans* significantly inhibited both cells by usnic acid (Figure 3G), whereas, without usnic acid treatment, the cells of *S. aureus* and *C. albicans* formed a mixed biofilm on the surfaces (Figure 3H). A similar result has also been observed in the case of the mixed-species biofilm of *S. mutants* with *C. albicans*, wherein the presence of the usnic acid, both cells have very little surface attached (Figure 3I), whereas, in the control, both cells form dense mixed-species biofilm (Figure 3J). The cells of *C. albicans* in the control group, combined with *S. aureus* and *S. mutans*, were present with some cell filamentous as bred in the mono-species control cells (Figure 3F).

## 3. Materials and Methods

### 3.1. Microbe, Chemicals, and Culture Media

Microbial pathogens such as *Candida albicans* (KCCM 11282), *Staphylococcus aureus* (KCTC 1916), and *Streptococcus mutans* (KCCM 40105) were used in this study. Tryptic soy broth (TSB), potato dextrose broth (PDB), and agar media were used for the cell cultivation of bacteria and fungal pathogens. The human artificial saliva (SAE0149-200 ML) was also purchased from Sigma-Aldrich (St. Louis, MO, USA). The composition of human artificial saliva has been reported previously [67].

### 3.2. Preparation of Titanium Coupons

The titanium sheet (commercially pure titanium, grade 2) with a thickness of 0.02 mm was cut into several small pieces, each with an area of 0.5 × 0.5 cm^2^. The titanium surface preparation was carried out as described earlier with slight modification [68]. Each coupon was cleaned by ultrasonicating it in ethanol for 30 min at 40 kHz. The coupons were sterilized with 95% ethanol, dried, and autoclaved.

### 3.3. Minimum Inhibitory Concentration (MIC) Determination

The MIC of *C. albicans*, *S. aureus*, and *S. mutants* was determined using the micro broth dilution technique. Each microbial culture was incubated for 12 h and then diluted (1:100) in their sterile growth media (TSB/PDB) [69]. The cultures were individually treated with usnic acid at 2048 µg/mL to 64 µg/mL concentrations) in a 24-well microplate, serially diluted, and further transferred to a 96-well polystyrene microplate in triplicates of 300 µL each. The microplates were incubated at 37 °C for 24 h without shaking. Further, the MIC was determined by visual inspection, and more than 90% of cell deaths were attained in treated samples. The optical density of cells at 600 nm (OD_600_) was also measured using a microplate reader. The MIC determination was repeated three times, and the experiment was conducted using triplicates.

### 3.4. Drug Combination for the Synergy Assays

Synergistic effects of antibiotics, antifungals, and usnic acid were tested in relationships with multiple bacterial microorganisms like *S. aureus*, *S. mutans*, and the fungal pathogen *C. albicans*. The interaction of usnic acid with standard antibiotics such as streptomycin, tetracycline, rifampicin, gentamycin, ciprofloxacin, and antifungal fluconazole, amphotericin B was investigated using the checkerboard broth dilution method [49,70]. For the process, sub-MIC (1/2-MIC) of antibiotics/ antifungal and the sub-MIC of usnic acid were added in the 96-well plate containing microbial cell culture (OD_600_ = 0.05) and carried out two-fold dilution. The micro-plate was incubated at 37 °C for 24 h, and the OD was determined to be at 600 nm.

The FIC calculation process and the summation of FIC were calculated as per the formula [70].

(1) $$\mathrm{FIC}\;\mathrm{index}=\frac{MICofUA\left(combinedMICofUA\right)}{MIC\left(UAalone\right)}+\frac{MICofdrug\left(combinedMICofdrug\right)}{MIC\left(drugalone\right)}$$

UA is the usnic acid, and drugs are antibiotics or antifungals. In order to evaluate the synergistic influence, the FICs were added together to produce the FIC index. The FIC index revealed distinct features depending on the index values, which were as follows: <0.5, synergic; >0.5 to ≤1, additive; >1 to ≤2, independent; >2, antagonistic [49].

### 3.5. Biofilm Inhibition Assays in the Standard Growth Media

The biofilm of mono- and mixed species of *S. aureus*, *S. mutans*, and *C. albicans* were carried out as described earlier [65]. For the mono-species biofilm assays, the cell culture of *S. aureus*, *S. mutans*, and *C. albicans*, each with an OD600 value of 0.05, was included in the 24-well microplate. Whereas, for the mixed-species biofilm assays, the equal volume of *S. aureus* (OD_600_ value of 0.05) and *C. albicans* (OD_600_ value of 0.05) prepared in their respective growth media and the same for the mixed-species biofilm of *S. mutans* and *C. albicans* were placed in the 24-well plate. Each well contained sterile titanium coupons (0.5 × 0.5 cm^2^) on which mono- and mixed species biofilm was allowed to develop in the presence of the standard growth media. These mono- and mixed-species cell cultures were also treated with sub-MIC of usnic acid. The untreated cells were considered the control group. The plates were incubated for 48 h at 37 °C. After growth, titanium plates were cleaned three times, immersed in 300 µL of TSB broth, sonicated (5 min at 40 kHz), and vortexed. This guaranteed that all connected biofilm cells were separated and submerged in TSB broth. The serial dilution was carried out up to 10^−8^ dilution, and the 100 µL culture was spread on the agar plate (TSA containing tetracycline and PDA plate containing fluconazole) to selectively allow the growth of the bacterial or fungal cells. After 24 h incubation at 37 °C, the colonies on the agar plate were enumerated, and the CFU value was determined.

### 3.6. Biofilm Inhibition Assay in Artificial Saliva Media

The overnight-grown seed cell cultures of *S. aureus*, *S. mutans*, and *C. albicans* were diluted in the artificial saliva with an OD_600_ value of 0.05 [71]. For the mono-species biofilm, these cells were placed in the 24-well containing titanium coupons. In the case of the mixed-species biofilm, an equal volume of the artificial diluted cell culture (OD_600_ value of 0.05) of bacteria with *C. albicans* was placed in the 24-well plate. These mono-species and mixed-species cell cultures were treated with sub-MIC of usnic acid. After incubation for 48 h at 37 °C, the cell enumeration was carried out in the same ways as discussed in the case of the biofilm assays in the standard growth media.

### 3.7. Microscopic Examination of Biofilms

Biofilms were grown on the surface of the titanium coupons for scanning electron microscopy (SEM) analysis to investigate the detachment inhibitory effects of usnic acid towards mono- and mixed-species biofilms in the artificial saliva [65]. The overnight-grown seed culture of *S. aureus*, *S. mutans*, and *C. albicans* in their respective growth media was diluted in the artificial saliva with an OD_600_ value of 0.05. These cells were put in the 24-well plates containing titanium coupons (0.5 × 0.5 cm^2^) for the mono-species biofilm. In the case of the mixed-species biofilm, an equal volume of the artificial saliva in diluted cell culture (OD_600_ value of 0.05) of bacteria with *C. albicans* was mixed, and the procedure was repeated with the 24-well plate containing titanium coupons. The sub-MIC of usnic acid was used to treat these mono- and mixed-cell cultures. The untreated cells were considered the control group. The plate was incubated at 37 °C for 48 h. Specific processes were performed to ensure biofilm attachment to the titanium coupons. Biofilm cells were subjected to formaldehyde (2%) and glutaraldehyde (2.5%) and cold-treated for 12 h at 4 °C. Then, we removed unattached cells by washing them three times with PBS (7.4), maintaining a cold temperature of 4 °C. Finally, they were exposed to increasing ethanol concentrations to facilitate moisture removal from cells safely. Then, the dry coupons were freeze-dried for 24 h, and the cells were visualized using field-emission scanning electron microscopy (JSM-IT800SHL, JEOL, Akishima, Japan).

### 3.8. Statistical Analysis

All data were statistically analyzed and plotted as the log mean value of CFUs and standard deviation (SD) of CFUs values using GraphPad Prism 7.0 (GraphPad Software Inc., San Diego, CA, USA). *** *p* < 0.0001, ** *p* < 0.01 and * *p* < 0.05 indicated significance. The log reduction values provided in the main text of the result were calculated as log reduction in CFU at different concentrations from the control group and shown as (mean log reduction values + standard deviation).

## 4. Conclusions

This study highlights the potential of usnic acid as an effective agent in controlling the growth of polymicrobial biofilms of bacterial and fungal pathogens on titanium surfaces in the host-mimicking media, which are commonly used for dental and orthopedic implants. When exposed to sub-MIC of usnic acid in artificial saliva, the results in this study demonstrated significant inhibition of biofilm-forming microorganisms, such as *S. aureus*, *S. mutans*, and *C. albicans* in both mono- and polymicrobial-species levels. Given that titanium, an effective and anticorrosive metal, can still experience degradation in the oral environment, due to prolonged exposure to an acidic environment generated by the metabolic activities of biofilm-forming microbial pathogens, usnic acid offers a promising solution to mitigate this issue. Furthermore, with the growing concern of antibiotic resistance due to high doses of conventional antibiotics, combination therapy incorporating usnic acid with antibiotics and antifungal agents shows substantial promise. The synergistic effects observed in this study suggest that usnic acid can enhance the efficacy of conventional antibiotics and antifungals towards bacteria and *C. albicans*, providing a more sustainable approach to managing biofilm-related infections in implant dentistry. This study supports the integration of usnic acid into therapeutic regimens for biofilm control, potentially reducing the risk of resistance and improving patient outcomes in implant-related procedures.

## 5. Limitations and Future Perspectives

Artificial saliva is widely used to simulate oral conditions, but it has limitations in drug efficacy testing. While it can mimic some properties of human saliva, such as pH and electrolyte composition [39], artificial saliva often fails to replicate the complex biocolloid nature and lubrication properties of real saliva [39,72]. Differences in viscosity, protein content, and surface tension can affect drug dissolution and absorption [73,74]. Additionally, artificial saliva lacks the natural antimicrobial defenses present in human saliva [75,76]. These limitations can lead to discrepancies between in vitro and in vivo results, highlighting the need for more physiologically relevant models [77,78]. To overcome these challenges, researchers are exploring advanced formulations and testing methods, including the use of human saliva and physiologically relevant irrigation media in ex vivo models [72].

In the present study, a host-mimicking media was used to determine the efficacy of usnic acid; however, the obtained efficacy and biofilm inhibition towards microbial pathogens have been determined in vitro conditions, which is the limitation of the present study. Hence, to further validate and recapitulate the antimicrobial action of usnic acid, future research will need to be carried out by performing in vivo experiments, using some animal model organisms.

Similarly, in vitro and in vivo studies have shown that surface roughness and hydrophilicity significantly influence bacterial adhesion and biofilm formation on titanium surfaces [79,80]. In vitro biofilm models using titanium surfaces are widely used but often fail to accurately represent in vivo conditions, limiting their clinical relevance [81,82]. These models typically lack the complexity of the host environment, including host-bacteria interactions and long-term dynamics [15,83]. In vivo biofilms differ significantly from in vitro biofilms in size, structure, and antimicrobial resistance [84]. To improve translatability, researchers suggest developing more complex models that better mimic the clinical scenario, incorporating tissue engineering concepts and considering the chemical microenvironment [81,85]. Recent efforts have focused on creating multifunctional titanium surfaces that promote osseointegration while preventing bacterial contamination [86,87]. However, these approaches still need optimization to address concerns such as modulating inflammatory responses and preventing prosthetic joint infections. In vitro models using constant-depth film fermentors and in vivo rabbit models have been developed to evaluate the effectiveness of different disinfection protocols for contaminated titanium surfaces [88]. These models provide valuable tools for studying peri-implantitis and developing effective prevention strategies.

## Figures and Tables

**Table 1:** MIC and FIC value of usnic acid, antibiotics, and antifungal agents in the standard growth media.

Combination of Drugs							Planktonic Cell Culture	
**Antimicrobial drugs**				**Usnic acid**			***S. mutans***	
**Name**	**MIC (µg/mL)**	**Combined MIC (µg/mL)**	**FIC**	**MIC (µg/mL)**	**Combined MIC (µg/mL)**	**FIC**	**ΣFIC**	**Interpretation**
ST	32	1	0.0313	8	0.063	0.063	0.0391	Synergy
TT	1	0.4790	0.4790	8	0.063	0.0079	0.4869	Synergy
RF	32	0.0310	0.0010	8	0.008	0.001	0.0020	Synergy
GT	16	0.5	0.0313	8	0.063	0.0079	0.0391	Synergy
CF	1	0.008	0.008	8	1	0.125	0.1330	Synergy
**Antimicrobial drugs**				**Usnic acid**			***S. aureus***	
**Name**	**MIC (µg/mL)**	**Combined MIC (µg/mL)**	**FIC**	**MIC (µg/mL)**	**Combined MIC (µg/mL)**	**FIC**	**ΣFIC**	**Interpretation**
ST	64	0.25	0.0039	16	0.125	0.0078	0.0117	Synergy
TT	1	0.016	0.016	16	1	0.0625	0.0785	Synergy
RF	1	ND	ND	16	ND	ND	ND	ND
GT	1	0.001	0.001	16	0.25	0.0156	0.0166	Synergy
CF	0.5	0.004	0.008	16	0.25	0.0156	0.023	Synergy
**Antimicrobial drugs**				**Usnic acid**			***C. albicans***	
**Name**	**MIC (µg/mL)**	**Combined MIC (µg/mL)**	**FIC**	**MIC (µg/mL)**	**Combined MIC (µg/mL)**	**FIC**	**ΣFIC**	**Interpretation**
FLC	128	0.125	0.0010	512	4	0.0078	0.0088	Synergy
AMB	1	0.004	0.004	512	2	0.0039	0.0079	Synergy

ND, not determined, ST—Streptomycin, TT—Tetracycline, RF—Rifampicin, GT—Gentamicin, CF—Ciprofloxacin, FLC—Fluconazole, AMB—Ampotericin B. Fractional inhibitory concentration (FIC) indexes (FICIs) of combined effects of antibiotics. Since there was no discernible difference in the MIC and FIC values of the replicates, only single values were given.

## Data Availability

Available on request.

## Abbreviation

AMR, antimicrobial resistance; UA, Usnic acid; CLSI, Clinical and Laboratory Standards Institute; PBS, phosphate-buffered saline; HAS, human artificial saliva; SEM, scanning electron microscopy; PDB/A, potato dextrose broth/agar; TSA, tryptic soy agar; TSB, tryptic soy broth; AmpB, amphotericin B; Tet, tetracycline.
